# Growth trajectory influences temperature preference in fish through an effect on metabolic rate

**DOI:** 10.1111/1365-2656.12244

**Published:** 2014-06-17

**Authors:** Shaun S Killen

**Affiliations:** 1 Institute of Biodiversity, Animal Health & Comparative Medicine, Graham Kerr Building, College of Medical, Veterinary & Life Sciences, University of GlasgowGlasgow, G12 8QQ, UK

**Keywords:** aerobic scope, compensatory growth, habitat choice, intraspecific variation, nutrition, physiological ecology

## Abstract

1. Most animals experience temperature variations as they move through the environment. For ectotherms, in particular, temperature has a strong influence on habitat choice. While well studied at the species level, less is known about factors affecting the preferred temperature of individuals; especially lacking is information on how physiological traits are linked to thermal preference and whether such relationships are affected by factors such feeding history and growth trajectory.
2. This study examined these issues in the common minnow *Phoxinus phoxinus*, to determine the extent to which feeding history, standard metabolic rate (SMR) and aerobic scope (AS), interact to affect temperature preference.
3. Individuals were either: 1) food deprived (FD) for 21 days, then fed *ad libitum* for the next 74 days; or 2) fed *ad libitum* throughout the entire period. All animals were then allowed to select preferred temperatures using a shuttle-box, and then measured for SMR and AS at 10 °C, estimated by rates of oxygen uptake. Activity within the shuttle-box under a constant temperature regime was also measured.
4. In both FD and control fish, SMR was negatively correlated with preferred temperature. The SMR of the FD fish was increased compared with the controls, probably due to the effects of compensatory growth, and so these growth-compensated fish preferred temperatures that were on average 2·85 °C cooler than controls fed a maintenance ration throughout the study. Fish experiencing compensatory growth also displayed a large reduction in activity. In growth-compensated fish and controls, activity measured at 10 °C was positively correlated with preferred temperature.
5. Individual fish prefer temperatures that vary predictably with SMR and activity level, which are both plastic in response to feeding history and growth trajectories. Cooler temperatures probably allow individuals to reduce maintenance costs and divert more energy towards growth. A reduction in SMR at cooler temperatures, coupled with a decrease in spontaneous activity, could also allow individuals to increase surplus AS for coping with environmental stressors. In warming climates, however, aquatic ectotherms could experience frequent fluctuations in food supply with long-lasting effects on metabolic rate due to compensatory growth, while simultaneously having limited access to preferred cooler habitats.

## Introduction

Nearly all animals are exposed to spatial and temporal variation in environmental temperature. For ectotherms in particular, environmental temperature can have a profound impact on various measures of performance including locomotion, growth and reproductive output (Huey & Kingsolver 1989; Angilletta, Niewiarowski & Navas 2002). In response to external temperature fluctuations or physiological demands, ectotherms often alter behaviours or select specific microhabitats to keep body temperature within a relatively narrow range (Reynolds 1978; Kearney, Shine & Porter 2009). While behavioural thermoregulation and thermal preferences of ectotherms have been well studied at the species level (Angilletta 2009), only a few studies have examined the extent to which individuals of the same species vary with respect to temperature preference (Pulgar *et al*. 2003; Stapley 2006; Clusella Trullas *et al*. 2007; Artacho, Jouanneau & Galliard 2013). In addition to being a basic requirement for natural selection, individual differences in behavioural and physiological traits are important determinants of how individuals and populations respond to changing environmental conditions (Killen *et al*. 2013; Humphries & McCann 2014).

It is possible that animals may show differences in thermal preference in relation to physiological traits (Angilletta, Niewiarowski & Navas 2002). Baseline metabolic demand [i.e. standard metabolic rate (SMR) in ectotherms] shows a large degree of variability among individuals of the same species (Burton *et al*. 2011) and varies with body temperature. It has previously been speculated that individuals with a relatively increased SMR at a given temperature will have increased foraging requirements to support their energetic demand and may therefore preferentially select cooler temperatures, particularly when food availability is low (van Dijk, Staaks & Hardewig 2002). This scenario is predicted by the allocation model of energy budgeting (Careau *et al*. 2008; or compensation model, Careau & Garland 2012), which states that basal metabolic demand competes with other physiological processes for acquired energy. Another potentially important physiological variable in determining thermal preference is absolute aerobic scope (AS), which is the difference between an individual's SMR and its maximal metabolic rate (MMR) and sets the capacity for simultaneous oxygen-consuming physiological tasks within an animal above maintenance requirements (e.g. growth, activity, digestion; Pörtner & Farrell 2008). Combined with the assumption that increased AS is associated with increased fitness (Brown, Marquet & Taper 1993), it has recently been suggested that AS has a large influence on the geographic distribution of ectothermic species (Pörtner & Knust 2007; Pörtner & Farrell 2008). Aerobic scope also shows variation within species (Norin & Malte 2011; Killen *et al*. 2012; Marras *et al*. 2013) that appears to be repeatable over at least short-and medium-term timeframes (Norin & Malte 2011) and could therefore influence individual temperature preference and habitat selection (Khan & Herbert 2012; Norin, Malte & Clark 2013). In contrast to the allocation model, which could generate a negative correlation between SMR and AS, the production model (Nilsson 2002; Careau *et al*. 2008) could result in a positive correlation between SMR and AS, because the ‘metabolic machinery’ (e.g. increased muscle mass, mitochondrial density) required to support an active lifestyle will also need to be maintained while at rest (Killen, Atkinson & Glazier 2010). This form of energy budgeting could generate positive correlations among SMR, AS and temperature preference, so that individuals capable of a high rates of energy throughput can fully realise this potential.

In addition to genetic sources of variation, environmental factors may generate variability among individuals with regard to temperature preference. Examples of such plasticity have been observed at the population level (Blouin-Demers *et al*. 2000; Mortensen, Ugedal & Lund 2007; Fangue *et al*. 2009), but environmental effects on temperature preference at the individual level have been mostly overlooked. Recent feeding, for example, may affect behavioural thermoregulation as individuals select temperatures which allow them to effectively digest their meal (Wallman & Bennett 2006). Over the longer term, there is evidence that fasting or energetically poor diets can decrease the preferred temperature of ectotherms (van Dijk, Staaks & Hardewig 2002; Pulgar *et al*. 2003) but the mechanisms that could underlie such a shift remain unknown. Dietary history could alter temperature preference by influencing growth trajectories and, by extension, SMR and available AS. For example, compensatory growth – the rapid growth that occurs after following a period of reduced growth – has been observed to cause a long-lasting alterations to numerous traits including locomotor ability (Álvarez & Metcalfe 2007), endurance during exercise (Royle, Lindstrom & Metcalfe 2006), and increased basal metabolic rate that persists even after the period of rapid growth has concluded (Criscuolo *et al*. 2008). Increased maintenance costs could cause a decrease in preferred temperature as individuals attempt to decrease maintenance requirements and increase the surplus energy available for growth (Priede 1985; Bryan, Kelsch & Neill 1990). An additional adverse effect of an increased SMR due to compensatory growth could be a decrease in available AS if there is no concomitant increase in MMR or offsetting changes in other energetically demanding processes, such as activity.

The aims of this study were to determine: (i) whether individual temperature preference is linked to physiological traits (i.e. SMR and AS) in individual animals; and (ii) whether the preferred temperature of individuals can be affected by growth trajectories, and specifically compensatory growth, either independently or via effects on physiological traits. I studied these questions in the common minnow *Phoxinus phoxinus*. This is a freshwater teleost fish that has a wide distribution across temperate zones in Europe in Asia. It is a habitat generalist, living in both flowing streams and lake environments where they are exposed to spatial and temporal fluctuations in thermal regimes. Although it feeds on a range of prey items (e.g. algae, detritus, macrobenthos), this species experiences seasonal variability in the quality and abundance of prey items and has been observed to exhibit compensatory growth following a period of food deprivation (Russell & Wootton 1992). Increased knowledge of the intrinsic and extrinsic factors that influence temperature preference in ectotherms will help us to better understand intraspecific variation in habitat use and how individuals will respond to aspects of environmental change.

## Materials and methods

### Experimental Animals

Wild minnows were caught in December 2011 from the River Endrick in Scotland using dip-nets and minnow traps. Fish were held in the laboratory at 10 °C for 3 weeks in two stock tanks (100 × 40 × 30 cm) before beginning experiments. During this time, fish were fed *ad libitum* using commercial trout feed. Fish were on a 12 h light: 12 h dark photoperiod during holding and throughout the experiment.

### Diet Treatments

**Figure 1:**
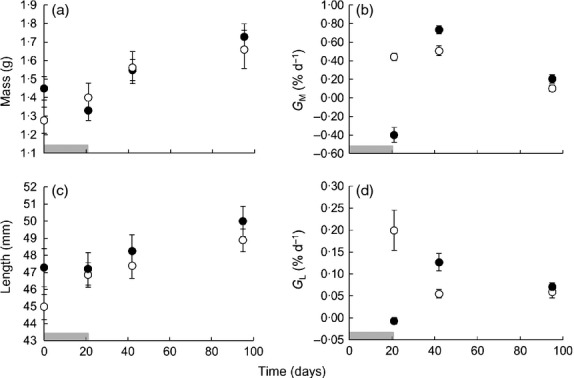
Growth trajectories in common minnows. Changes in: (a) total wet mass; (b) mass-specific growth rate (*G*_M_); (c) standard length; and (d) length-specific growth rate (*G*_L_) in common minnows fed either *ad libitum* throughout the entire 95-day study (control treatment; open circles), or food deprived for 21 days before being fed *ad libitum* for the remainder of the study [food-deprived (FD) treatment; dark circles]. In panels b and d, data points represent growth rates up until the day on which they are plotted, from the previous day point. Horizontal shaded bars represent the duration of food deprivation in the FD treatment.

Fish were netted from stock tanks then lightly anaesthetised, weighed (±0·001 g) and photographed for later measurement of standard length (imageJ, Schneider, Rasband & Eliceiri 2012). After recovery, individual fish were placed into 10-L tanks (one fish per tank; Russell & Wootton 1992), each containing a plastic plant and small filter that also bubbled air into the water. Tanks were filled with dechlorinated tap water. Waste was siphoned from the tanks daily with 30% of the water being changed each week. Tanks were randomly assigned to one of two treatments (*n* = 13 fish in each treatment): (i) a control group, in which fish were fed *ad libitum* with commercial feed for the first 21 days of the study; or (ii) a food-deprived (FD) group, in which fish were completely deprived of food for the first 21 days of the study, referred to as the ‘fasting phase’. Although there was a small initial difference in length and mass between the groups when the fish were randomly assigned to the treatments, this difference was not significant (general linear models, effect of treatment, *P* > 0·05; Fig.1). Temperate fish can readily withstand prolonged periods of fasting when at low ambient temperatures, and in degree days (water temperature × time in days), the period of fasting used in the current study was close to that previously used to study the effects of fasting on subsequent growth trajectories in common minnows (21 days × 10 °C = 210 degree days in the current study compared with 16 days × 15 °C = 240 degree days in Russell & Wootton 1992). After this point, both treatments were fed *ad libitum* for the next 74 days (Álvarez & Metcalfe 2007), referred to as the ‘growth phase’. Fish were again anaesthetised, measured and photographed on days 21 (at the end of the fasting phase), 42, and 95 of the study.

### Temperature Preference

At the end of the 95-day diet treatments, temperature preference was tested in individual fish using a shuttle-box apparatus (Loligo Systems, Tjele, Denmark) in which the environmental temperature experienced by the animal is controlled by its behaviour (Petersen & Steffensen 2003). The shuttle-box consisted of two opaque circular plastic choice chambers (40 cm diameter) joined at the centre of one edge by a passage way (10 cm) to form a ‘dumb-bell’ shape. Each choice chamber contained water to a depth of 7 cm and was attached by silicone tubing to its own dedicated external buffer tank. One of the choice chambers was designated as the warm chamber and the other as the cold one. External to both the choice chambers and the buffer tanks were a heating reservoir and a cooling reservoir. The heating reservoir was supplied with an electric aquarium heater, while the chilling reservoir was cooled with an external chilling unit. Water was pumped from the buffer tanks and through steel coils in either reservoir to alter the temperature within each buffer tank as required.

The temperature within each of the choice chambers was continually monitored using in-line temperature probes. The temperature probes were connected to a computer-driven temperature controller and data acquisition system (DAQ-M; Loligo Systems). The temperature within each choice chamber was ultimately controlled by software (shuttlesoft; Loligo Systems) which, via a video display from a camera (uEye; Imaging Development Systems GmbH, Obersulm, Germany) mounted above the tank, detected the position of the fish and adjusted flow from the reservoir tanks to change the temperature in the choice chambers accordingly. Specifically, the temperature within either choice chamber could be set to be either in a static or in dynamic mode. When ‘static’, there was a constant temperature in each choice chamber but a 2 °C differential between them. When the system was ‘dynamic’ the temperature would change within the shuttle-box depending on the location of the fish, while maintaining a 2 °C differential between the warm and cold chambers. Thus if a fish moved into the warm chamber, the temperature would increase in both sides of the shuttle-box at a rate of 2 °C h^−1^ (to a pre-set maximum of 24 °C), but the warm chamber would always remain 2 °C warmer than the cold chamber. Conversely, when a fish moved to the cold chamber, the temperature in both sides of the shuttle-box would begin to decrease by 2 °C h^−1^ (to a pre-set minimum of 3 °C). Thus, by alternating between the warm and cold chambers of the shuttle-box in response to changing temperatures, an individual could regulate the environmental temperature that it experienced. Pilot experiments demonstrated a high repeatability for final preferred temperatures in common minnows using this system over a 1-week period at the same acclimation temperature (*n* = 10 individuals, Pearson correlation between trials, *r* = 0·89, *P* = 0·0005).

During each testing run, a single fish that had been fasted for 24 h was placed in the shuttle-box that had been set to the static mode, with the warm chamber at 11 °C and the cold chamber at 9 °C. The fish was then left overnight. The next morning, activity within the shuttle-box (total distance moved in cm) was calculated by tracking the *x*−*y* coordinates of the fish (once per second) with the mounted camera and associated software between the hours of 08:00–10:00. At this point, the system was switched to the dynamic mode, and the fish was left to select its preferred temperature for the next 8 h. Core body temperature was estimated once every second using the following equation: 

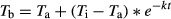
eqn 1

where *T*_b_ is core body temperature, *T*_a_ is ambient water temperature, *T*_i_ is initial temperature, *t* is time between temperature readings (min), and *k* is the rate of change of core temperature, which varies with body size. A specific value for *k* is not available for the common minnow, and so *k* was approximated using the relationship between *k* and mass (*m*) for another freshwater teleost, the white sucker *Catostomus commersoni* (Stevens & Fry 1974): 

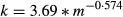
eqn 2

The mean core body temperature experienced during the final 2 h was taken as the final preferred temperature of each individual. By estimating core body temperature, rather than simply taking the water temperature, the final preferred temperature is less likely to be influenced by brief excursions of fish into alternate chambers for temperature ‘sampling’. Following the temperature selection protocol, the fish was then returned to its individual tank and replaced with another individual, to be tested the following day. No food was provided during the period spent in the shuttle-box.

### Measurement of Metabolic Rate and Aerobic Scope

Following temperature choice trials (4–7 days after), metabolic rates were estimated for each fish as rates of oxygen uptake using intermittent stopped-flow respirometry (Steffensen 1989; Killen *et al*. 2012). Each day at approximately 10:00 h, three fish that had been fasted for 24 h were placed into individual cylindrical 110-mL PCV respirometers and left undisturbed overnight. One respirometer was left empty to control for background bacterial oxygen consumption. For all measurements, water oxygen content was quantified once every 5 s using a Firesting 4-channel oxygen meter and associated sensors (PyroScience GmbH, Aachen, Germany). The respirometers were located within an aerated, rectangular, temperature regulated water bath (10 °C; 11·5 L) and were shielded from external disturbance and direct lighting by an opaque plastic blind. Water mixing within each respirometer was achieved with a peristaltic pump that moved water through the chamber and round an external circuit of gas-impermeable tubing. Every 15 min an automated flush pump would switch on or off. When off, the respirometers were sealed and the decrease in oxygen content could be analysed to indicate rate of oxygen uptake. When open, the respirometers would be flushed with aerated water. Lighting was set to turn on at 07:00 h and off at 19:00 h. Fish were removed from the respirometer at around 08:00 h the following day, having remained in the respirometers for approximately 22 h in total. Whole-animal SMR (SMR; in mg O_2_ h^−1^) was estimated as the lowest 10th percentile of measurements taken throughout the measurement period (Dupont-Prinet *et al*. 2010; Killen *et al*. 2012), excluding the first 5 h of confinement in the chambers during which oxygen consumption was often increased. Routine metabolic rate (RMR; mg O_2_ h^−1^) was measured as the mean level of oxygen uptake during this time.

At this stage, fish were removed from the respirometers and subjected to exhaustive exercise by manually chasing the fish in a circular tank (50 cm diameter) with a water depth of 10 cm (Killen *et al*. 2012; Clark, Sandblom & Jutfelt 2013). After complete exhaustion, which occurred within 3–5 min of chasing, fish were immediately returned to the respirometers. Time between the cessation of swimming and closure of the respirometer was always <15 s. Rates of oxygen uptake were then measured in 3-min intervals during a 15-min closed phase in the respirometers, and the maximal rate of oxygen uptake measured during this time was taken as MMR (in mg O_2_ h^−1^). This method assumes that maximal rates of oxygen uptake are achieved during the recovery from the bout of exhaustive anaerobic exercise (e.g. as stores of glycogen and ATP are replenished), and in several fish species, this method has been demonstrated to elicit rates of oxygen uptake equal to or greater than that observed during sustained swimming (Reidy *et al*. 1995; Killen *et al*. 2007; Clark, Sandblom & Jutfelt 2013).

### Data and Statistical Analyses

Statistics were performed with spss statistics v20.0 (SPSS Inc. and IBM, Chicago, IL, USA) and sigmaplot v11.0 (Systat Software Inc., San Jose, CA, USA). The level of significance for all tests was α = 0·05. When required the normality, linearity and homoscedasticity of residuals were verified by inspection of residual-fit plots, with particular attention paid to homoscedasticity of variances between diet treatments (Cleasby & Nakagawa 2011). For use in models, measures of SMR, RMR, MMR, AS, mass and activity were log-transformed.

Rates of mass loss or growth were estimated in terms of body mass (*G*_M_) and standard length (*G*_L_) and were calculated between measurement periods according to the equation: 

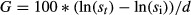
eqn 3

where *s*_*t*_ is the body mass or standard length at time *t*,*s*_i_ is the initial body mass or standard length and *d* is the time elapsed in days (Hopkins 1992). Growth rates of control and FD treatments were compared during the starvation phase using general linear models with growth (either *G*_M_ or *G*_L_) as the dependent variable, treatment as a categorical variable and either mass (for *G*_M_) or standard length (*G*_L_) at the beginning of each measurement period as a covariate. During the growth phase, repeated measures were accommodated using a linear mixed model with fish identity added as a random factor and measurement period as a repeated factor.

Measures of SMR, MMR, AS, factorial aerobic scope and activity level were compared between control and FD treatments using general linear models with treatment as a categorical variable. Mass was also included as a covariate to correct for the effects of body size. Changes in body temperature during shuttle-box trials (means taken at each 30-min intervals throughout the 8-h trial) were compared between treatments using a linear mixed model with random intercepts and slopes with diet treatment as a categorical variable, mass as a covariate, fish identity as a random factor and time as a repeated factor. A quadratic term (time^2^) was also added to the model to account for the curvilinear nature of the change in mean body temperature with time during each interval (Sokal and Rohlf 1995). The inclusion of random slopes accounted for any individual differences in changes in preferred temperature with time. Initial models also included the interactions between diet and time, and diet and time^2^.

Effects of SMR, AS, *G*_L_ during the growth phase, and activity on final preferred temperature were initially explored with Pearson correlations. General linear models were used to examine the combined effects of these variables on the final preferred temperature, with treatment as a categorical variable, and SMR, AS, *G*_L_ during the growth phase, activity, and mass as continuous variables, as well as interactions between each factor and diet treatment. In all models, non-significant interactions were sequentially dropped and the models re-run.

## Results

### Effects of Food Deprivation on Growth Rate

During the food-deprivation phase, treatments differed in both *G*_L_ and *G*_M_ (Fig.1; general linear model, effect of treatment on *G*_L_: *F*_1,25_ = 18·638, *P* < 0·001; *G*_M_: *F*_1,25_ = 85·549, *P* < 0·001). During the growth phase, FD fish gained mass and length more rapidly than controls after accounting for differences in body size at the beginning of each measurement period (linear mixed models, treatment effect on *G*_L_: *F*_1,25_ = 4·322, *P* = 0·044; *G*_M_: *F*_1,25_ = 11·199, *P* < 0·002).

### Effects of Diet on Metabolic Phenotype

**Figure 2:**
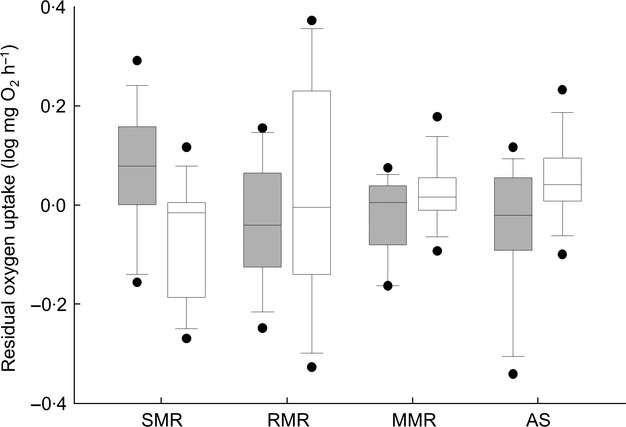
Metabolic traits for common minnows fed a control diet or that experienced earlier food deprivation. Control minnows were fed *ad libitum* throughout the entire 95-day study (control treatment; grey boxes), while food-deprived fish were fasted for 21 days before being fed *ad libitum* for the remainder of the study (food-deprived treatment; dark boxes). SMR, standard metabolic rate; RMR, routine metabolic rate; MMR, maximal metabolic rate; AS, aerobic scope. Values are residuals after correction for body size; see Fig. S1 (Supporting Information) for raw per animal data (mg O_2_ h^−1^)

At the end of the 95-day study, the whole-animal SMR of FD fish was on average 51·9% higher than that of fish in the control group and remained significantly increased after accounting for individual variation in body mass (Fig.2; general linear model, effect of treatment: *F*_1,25_ = 9·697, *P* = 0·005). While values for RMR showed an opposite trend, with RMR being 19·1% higher in control fish as compared with FD fish, this difference was not significant after correcting for mass (general linear model, *F*_1,25_ = 0·778, *P* = 0·384). Whole-animal MMR of control fish was 8·08% higher than that of FD fish, but this difference was also not significant (general linear model, *F*_1,25_ = 3·021, *P* = 0·096). However, control fish had values for AS that were on average 19·8% higher than for FD fish (general linear model, *F*_1,25_ = 5·416, *P* = 0·029). Factorial scope was also significantly lower in FD fish (5·25 ± 0·64) as compared to controls (8·08 ± 0·71; general linear model, *F*_1,25_ = 4·923, *P* = 0·037). FD fish were much less active than control fish in the shuttle-box apparatus when held at the two static temperatures, moving on average 59·97% of the total distance moved by control fish (general linear model, effect of treatment: *F*_1,25_ = 14·753, *P* = 0·001). Among individuals, activity was not related to either SMR or AS (general linear models, *P* > 0·05). Overall, there was a negative correlation between SMR and AS among individuals (Fig. S2, Supporting Information; Pearson correlation, *r* = −0·646, *P* = 0·0004).

### Temperature Preference

**Figure 3:**
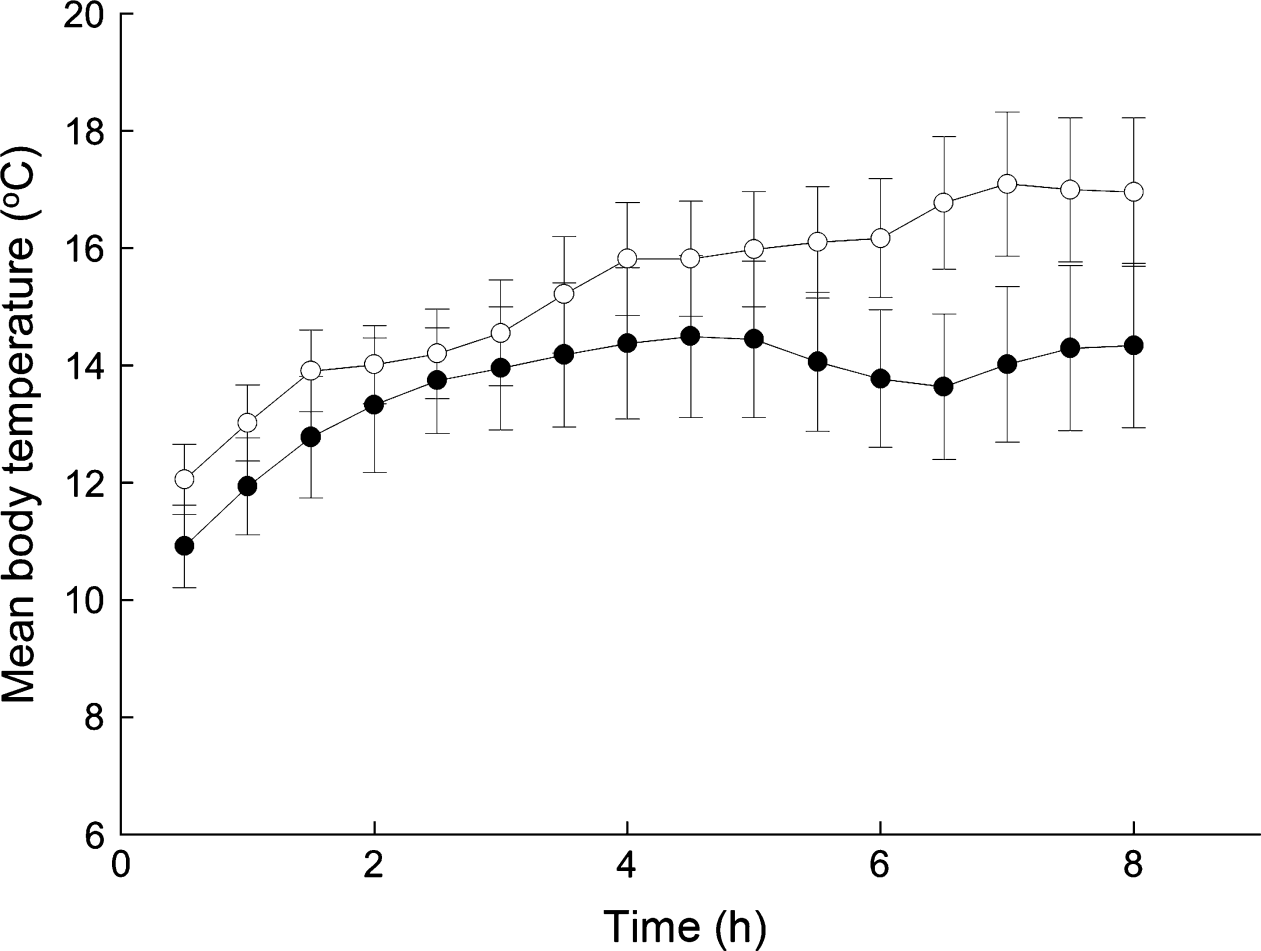
Mean body temperatures for common minnows held in a shuttle-box apparatus in individuals could select their preferred temperature. Values at each point were calculated as the mean temperature during a given 30-min interval. Treatments were fed either *ad libitum* throughout the entire 95-day study (control treatment; open circles), or food deprived for 21 days before being fed *ad libitum* for the remainder of the study (food-deprived treatment; dark circles). Error bars = SEM.

During the 8-h dynamic temperature selection period, fish in both treatments tended to shift toward temperatures warmer than the 10 °C acclimation temperature (Fig.3; linear mixed model, effect of time: *F*_1,25_ = 11·184, *P* = 0·003). However, throughout this time FD fish preferred colder temperatures than controls (linear mixed model, effect of treatment: *F*_1,345·93_ = 11·306, *P* = 0·001), with the mean final preferred core body temperature of the FD group (13·94 ± 1·27 °C) being over two degrees lower than that of the control group (16·79 ± 1·16 °C). Interactions between diet treatment and both of time and time^2^ were not significant (linear mixed model, treatment × time and treatment × time^2^ interactions, *P* > 0·05) and were not included in the final model.

**Figure 4:**
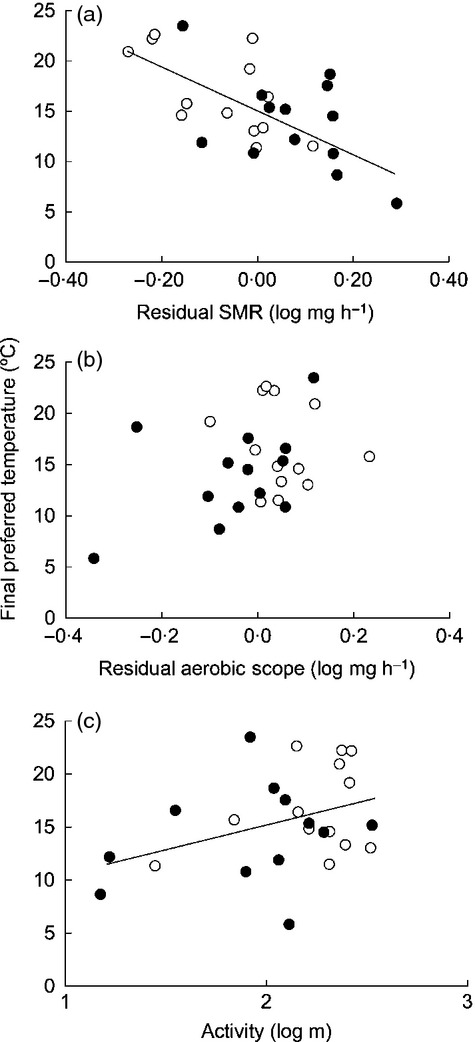
Relationships between preferred body temperature in individual minnows and metabolic and behavioural traits. (a) standard metabolic rate (SMR); (b) aerobic scope (AS); and (c) activity, in common minnows fed either *ad libitum* throughout the entire 95-day study (control treatment; open circles), or food deprived for 21 days before being fed *ad libitum* for the remainder of the study (food-deprived treatment; dark circles). Each point represents one individual. Solid regression lines represent the significant main effects of SMR (panel a) and activity (panel c) on final preferred temperature described in Table1. There was no effect of aerobic scope (panel b) on preferred temperature.

When all potential explanatory variables were combined into a single model, final temperature preference was influenced by SMR, with individuals that had a higher SMR preferring cooler temperatures (Fig.4; Table1; general linear model, effect of SMR: *F*_1,25_ = 9·076, *P* = 0·007). Individuals that were more active tended to prefer warmer temperatures (Fig.4; Table1; effect of activity: *F*_1,25_ = 4·469, *P* = 0·049). Although diet treatment was linked to temperature preference when examined independently (Fig.3), it did not have a significant effect on final preferred temperature once SMR and activity were taken into account (Table1). AS did not have a significant effect on temperature preference (Table1; Fig.4).

**Table 1:** General linear model results for effects on preferred temperature in common minnows acclimated to 10 °C, *n* = 13 per diet treatment

Term	d.f.	Mean square	*F*	*P*	Parameter	SEM
Diet	1	16·449	1·197	0·288	−2·522	2·192
SMR	1	124·698	9·076	0·007	−21·785	7·487
AS	1	22·417	1·632	0·218	−0·015	8·781
Activity	1	66·956	4·469	0·049	3·771	2·621
*G*_L_	1	2·632	0·192	0·667	−11·858	27·092
Mass	1	21·645	1·575	0·225	5·933	4·845
Error	18	11·291				

SMR, standard metabolic rate; AS, absolute aerobic scope; *G*_L_, growth rate during the growth phase (days 21–95 of the current study).

Non-significant two-way interactions between diet treatment and other factors were dropped from the model.

## Discussion

Individuals that experienced earlier food deprivation and subsequent compensatory growth tended to prefer cooler temperatures, but the underlying cause of this link is the effect of compensatory growth on SMR. Thus while compensatory growth resulted in an increase of SMR several weeks after the resumption of normal feeding, even individuals in the control treatment with a relatively high SMR preferred cooler temperatures. As a consequence, when all potential explanatory variables were included in the same model, only SMR and activity level were revealed to be important for influencing thermal preference. Overall, these results demonstrate that metabolic demand influences the thermal preferences of individual fish, and that preferred temperature is plastic, being affected by factors such as compensatory growth.

During the growth phase, FD individuals displayed increased growth rate in terms of length as well as mass, indicating they were experiencing increased rates of structural growth as opposed to simply restoring lost mass (Nicieza & Álvarez 2009). It is likely that fasted fish were undergoing compensatory growth during the growth phase of the study, as has been previously demonstrated to occur in common minnows after a prolonged period of food deprivation (Russell & Wootton 1992). In the current study, fish that had experienced compensatory growth had relatively increased levels of SMR many weeks after food became freely available; a similar long-lasting effect on metabolic rate of an earlier period of compensatory growth has been previously noted in other taxa (Criscuolo *et al*. 2008). Decreased tissue quality stemming from rapid growth could subsequently lead to increased rates of protein turnover or repair (Johnston 1999), so increasing rates of maintenance metabolism (Houlihan 1991). It may also be possible that altered growth trajectories can affect the allometry of organ sizes and changes in body composition that cause differences in the relative sizes of metabolically active organs and tissues (e.g. liver, muscle) in growth-compensated individuals (Oikawa, Takemori & Itazawa 1992).

By selecting a cooler temperature, individuals with a relatively high SMR could increase growth efficiency by decreasing baseline maintenance costs (Ware 1982; Mehner & Wieser 1994). For the FD treatment, this effect of SMR on thermal preference may have been exaggerated as they were observed to have an increased SMR as compared to the control individuals. This finding is compatible with the allocation model of energy budgeting (Careau *et al*. 2008), in which increased maintenance costs necessarily detract from the energy available for additional physiological functions, possibly including growth. Added support for this hypothesis is the observation that, at 10 °C, there was a negative correlation between SMR and AS. This indicates that individuals with a relatively high SMR do not compensate with a correspondingly high MMR, and thus will face greater trade-offs among oxygen-demanding physiological tasks compared with those with a lower SMR.

An additional benefit of a reduced SMR, however, is an increase in surplus AS for coping with conditions that may arise which increase baseline metabolic costs or that limit MMR (masking and limiting factors in Fry 1971; for example disease, environmental stressors). Animals that operate near MMR experience an increased likelihood of mortality (Wood, Turner & Graham 1983; Priede 1985), and there should be selection to prefer environments which increase surplus AS after accounting for the costs of activity and digestion (Ware 1982; Priede 1985; Bryan, Kelsch & Neill 1990). Decreasing SMR (by selecting cooler waters) and also reducing other energetic costs – such as that due to activity – could allow an animal to engage in growth while maintaining a surplus of available AS. Importantly, a maximum surplus of *available* AS could be attained below the theoretical optimum temperature for *total* AS (*sensu* Pörtner & Farrell 2008). This is because increased SMR at higher temperatures necessitates increased foraging and the associated costs of digestion and activity, thus diminishing the surplus AS actually available for accommodating limiting and masking factors (Fry 1971). This likely explains the reduced activity observed among FD fish several weeks after food became freely available. Indeed, even though SMR was different between FD and control fish, residual RMR (which includes the costs of spontaneous activity) was equal between the two treatments. This suggests that FD fish effectively offset increased maintenance costs by decreasing activity. It is important to note that fish in the current study were measured for metabolic traits at a constant intermediate temperature (10 °C), while they chose to occupy a wide range of temperatures (*c*. 6–23 °C). Assuming the original temperature is at or below that which optimises AS, a further decrease in temperature will cause a drop in both MMR and SMR (Claireaux & Lefrancois 2007; Pörtner & Farrell 2008). All other factors being equal, total AS should therefore remain unchanged or more likely decrease as temperatures cool (depending on the relative thermal sensitivities of SMR and MMR over the temperature range in question). However, by modulating other energetic costs, such as activity, individuals could increase surplus AS relative to that available at a warmer temperatures, better accommodating growth and increasing the capacity to deal with environmental stressors.

It has been hypothesised that a primary driver for habitat selection in aquatic ectotherms may be the optimisation of AS (Fry 1971; Pörtner & Farrell 2008; Jørgensen *et al*. 2012). The current study detected no significant main effect of AS on temperature preference. It is possible that increased statistical power would reveal such an effect, but even in this case, the effect size of SMR on thermal preference exceeds that of AS (Table1). Interestingly, preferred temperatures among and within species do not always coincide with the optima for specific physiological traits (Angilletta 2009; Schram *et al*. 2013). In addition to increasing surplus AS by minimising SMR and other energetic costs, a non-mutually exclusive possibility is that different individuals prioritise different functions (e.g. swim performance, immune function), with each function having its own thermal optimum (Clark, Sandblom & Jutfelt 2013). Alternatively, some individuals may simply minimise SMR during behavioural thermoregulation, as opposed to the optimisation of specific traits. Again, an important consideration is that fish in the current study were measured for SMR and MMR at a single temperature. It is possible that individuals vary in thermal sensitivity, thus altering the relative ranking of metabolic traits when exposed to different temperatures. More work is needed to undertake the challenging tasks of measuring how SMR and AS dynamically shift as individuals move through the environment and are exposed to spatial and temporal variation in thermal regimes (Norin, Malte & Clark 2013) and to examine the thermal stability of repeatability for traits such as SMR and AS within populations. A key area for future research will be determining the extent to which individual temperature preference overlaps with the temperature that optimises total AS vs. that which maximises surplus AS actually available after the costs of required processes such as activity and digestion are accounted for (which will vary across temperatures).

At the time of measurement for SMR and AS, FD fish had ‘caught up’ to control fish in terms of size, and *G*_L_ was not increased at this time. Additional research is needed to examine the thermal preference of individuals actively engaging in extremely rapid or compensatory growth. Under these circumstances, there may be a positive correlation between SMR and preferred temperature, as growth rate in fishes is often positively correlated with temperature (until an optimum at which maximum growth rate is achieved; Jobling 1995). Even in this scenario, however, maximising growth without countering mechanisms to balance the surplus AS (e.g. by reducing activity) could constrain the ability to cope with environmental stressors (Priede 1985; Bryan, Kelsch & Neill 1990), and so cooler temperatures may still be preferred when possible. Indeed, van Dijk, Staaks & Hardewig (2002) observed that immediately following a period of starvation, juvenile roach *Rutilus rutilus* preferred cooler temperatures even while displaying evidence of a compensatory growth response. The results of the present study confirm that this preference for cooler temperatures is related to maintenance metabolism in individual fish, even in those not undergoing compensatory growth, and is likely a strategy for increasing growth efficiency and a surplus of available AS for additional oxygen-consuming functions.

Although relationships between dietary history, AS, and temperature preference are underpinned by variation in SMR, these links are still likely to be of key ecological importance. Relatively warm microclimates can be beneficial for ectothermic individuals that can afford to occupy them (i.e. those with a low SMR or high AS), as these facilitate increased activity and rates of foraging. However, if earlier perturbations to growth cause an increase in SMR then these microclimates will be less suitable. Future changes in climate are predicted to cause increased fluctuations in food availability in temperate freshwater habitats (Winder & Schindler 2004). This phenomenon could increase the occurrence of compensatory growth among freshwater ectotherms while at the same time any environmental warming may reduce the availability of preferred microhabitats. Further, effects on activity levels stemming from compensatory growth could have profound implications for the behavioural ecology of fish, possibly affecting foraging ability or predator avoidance (Álvarez & Metcalfe 2007).

Fish that were more active at 10 °C tended to prefer warmer temperatures, and it is possible that, in conjunction with variation in physiological traits, variation in behavioural tendencies also influence temperature preference. Activity has previously been noted as an intrinsic trait within individuals of the same species (Careau *et al*. 2008), and if the shuttle-box is treated as a novel environment, then at least a portion of this activity may have also been due to exploration, which is also considered a dimension of animal personality (Dingemanse *et al*. 2002). The results here show that these personality traits can be plastic in response to factors such as growth trajectory and dietary history (Méndez & Wieser 1993). However, in addition to metabolic traits, differing personality types may cause individuals to have differing strategies when it comes to temperature selection, opting to either occupy cooler habitats that minimise energy expenditure (less active individuals) or relatively warmer habitats that facilitate a more active lifestyle or maximise AS (those that are more active). In support of this view, Stapley (2006) observed that more aggressive male lizards preferred warmer temperatures. Activity and boldness may also be positively correlated among individuals (Réale *et al*. 2010), and so bolder and more active individuals may be more willing to occupy warmer areas and accept the risks associated with increased rates of foraging that would be required to support a higher SMR in these environments. Interestingly, it has previously been hypothesised that more active individuals may also possess increased resting metabolic rates, because the morphology needed to support high levels of activity (e.g. increased muscle mass, mitochondrial density) will need to be maintained even at rest (the production or increased intake models of Nilsson 2002; Careau *et al*. 2008; Careau & Garland 2012). However, the lack of an association between SMR and activity in the current study instead provides additional support for the allocation model of energy budgeting (Careau *et al*. 2008).

In summary, the results of this study suggest that fish that have gone through a period of compensatory growth tend to prefer cooler temperatures, and that these effects are mediated through an increase of SMR, which reduces the scope for aerobic metabolism. An additional avenue for future research will be to examine how such shifts in temperature preference interact with other environmental variables in the wild, such as predator risk and food availability.

## Data accessibility

Data available from the Dryad Digital Repository: http://doi.org/10.5061/dryad.5h2sd (Killen 2014).

## Supporting Information

Fig. S1. Metabolic traits for common minnows fed a control diet or that experienced earlier food deprivation.

Fig. S2. Relationship between residual standard metabolic rate (SMR) and residual aerobic scope among common minnows at 10 °C, after correction for body mass.
