# Supplementary material for: Growth trajectory influences temperature preference in fish through an effect on metabolic rate
Source: J Anim Ecol. 2014 Jun 17;83(6):1513–22. doi: 10.1111/1365-2656.12244 (PMC4277333; doi:10.1111/1365-2656.12244)
Supplement: Supplementary file 1 — Fig. S1. Metabolic traits for common minnows fed a control diet or that experienced earlier food deprivation. [file jane0083-1513-sd1.docx]

**FIGURE s1.** Metabolic traits for common minnows fed a control diet or that experienced earlier food-deprivation. Control minnows were fed *ad libitum* throughout the entire 95 day study (control treatment; grey boxes), while food deprived fish were fasted for 21 days before being fed *ad libitum* for the remainder of the study (food-deprived treatment; dark boxes). * indicates a significant difference between the control and food-deprived treatments (general linear models, p < 0.05, see Results for details). SMR = standard metabolic rate; RMR = routine metabolic rate; MMR = maximal metabolic rate; AS = aerobic scope.
