# Supplementary material for: Growth trajectory influences temperature preference in fish through an effect on metabolic rate
Source: J Anim Ecol. 2014 Jun 17;83(6):1513–22. doi: 10.1111/1365-2656.12244 (PMC4277333; doi:10.1111/1365-2656.12244)
Supplement: Supplementary file 2 — Fig. S2. Relationship between residual standard metabolic rate (SMR) and residual aerobic scope among common minnows at 10 °C, after correction for body mass. [file jane0083-1513-sd2.docx]

**FIGURE s2.** Relationship between residual standard metabolic rate (SMR) and residual aerobic scope among common minnows at 10^o^C, after correction for body mass. Each data point is one individual. Fish were fed either *ad libitum* throughout the entire 95 day study (control treatment; open circles), or food deprived for 21 days before being fed ad libitum for the remainder of the study (food-deprived treatment; dark circles). Solid line represents thelinear regression across both treatments (residual aerobic scope = -0.000024 + (-0.53 * residual SMR), r^2^= 0.42, p = 0.0004).
